# The Use of Mobile Technologies to Promote Physical Activity and Reduce Sedentary Behaviors in the Middle East and North Africa Region: Systematic Review and Meta-Analysis

**DOI:** 10.2196/53651

**Published:** 2024-03-19

**Authors:** Huong Ly Tong, Aroub Alnasser, Najim Z Alshahrani, Rowaedh A Bawaked, Reem AlAhmed, Reem F Alsukait, Severin Rakic, Volkan Cetinkaya, Hazzaa M Al-Hazzaa, Saleh A Alqahtani

**Affiliations:** 1 Cultural and Indigenous Research Centre Australia Redfern Australia; 2 The World Bank Group Washington, DC United States; 3 Department of Food Science and Nutrition King Saud University Riyadh Saudi Arabia; 4 Department of Family and Community Medicine Faculty of Medicine University of Jeddah Jeddah Saudi Arabia; 5 Department of Public Health Saudi Electronic University Riyadh Saudi Arabia; 6 Department of Biostatistics, Epidemiology and Scientific Computing King Faisal Specialist Hospital & Research Center Riyadh Saudi Arabia; 7 Health Sciences Research Center Princess Nourah Bint Abdulrahman University Riyadh Saudi Arabia; 8 Organ Transplant Center of Excellence King Faisal Specialist Hospital & Research Center Riyadh Saudi Arabia; 9 Division of Gastroenterology & Hepatology Johns Hopkins University Baltimore, MD United States

**Keywords:** mobile apps, fitness trackers, SMS, SMS text messaging, physical activity, exercise, sedentary behavior, Middle East, Africa, Northern, movement, physical inactivity, smartphone, mobile phone, mobile health, mHealth, digital health, behavior change, intervention

## Abstract

**Background:**

The Middle East and North Africa (MENA) region faces unique challenges in promoting physical activity and reducing sedentary behaviors, as the prevalence of insufficient physical activity is higher than the global average. Mobile technologies present a promising approach to delivering behavioral interventions; however, little is known about the effectiveness and user perspectives on these technologies in the MENA region.

**Objective:**

This study aims to evaluate the effectiveness of mobile interventions targeting physical activity and sedentary behaviors in the MENA region and explore users’ perspectives on these interventions as well as any other outcomes that might influence users’ adoption and use of mobile technologies (eg, appropriateness and cultural fit).

**Methods:**

A systematic search of 5 databases (MEDLINE, Embase, CINAHL, Scopus, and Global Index Medicus) was performed. Any primary studies (participants of all ages regardless of medical condition) conducted in the MENA region that investigated the use of mobile technologies and reported any measures of physical activity, sedentary behaviors, or user perceptions were included. We conducted a narrative synthesis of all studies and a meta-analysis of randomized controlled trials (RCTs). The Cochrane risk-of-bias tool was used to assess the quality of the included RCTs; quality assessment of the rest of the included studies was completed using the relevant Joanna Briggs Institute critical appraisal tools.

**Results:**

In total, 27 articles describing 22 interventions (n=10, 37% RCTs) and 4 (15%) nonexperimental studies were included (n=6141, 46% women). Half (11/22, 50%) of the interventions included mobile apps, whereas the other half examined SMS. The main app functions were goal setting and self-monitoring of activity, whereas SMS interventions were primarily used to deliver educational content. Users in experimental studies described several benefits of the interventions (eg, gaining knowledge and receiving reminders to be active). Engagement with the interventions was poorly reported; few studies (8/27, 30%) examined users’ perspectives on the appropriateness or cultural fit of the interventions. Nonexperimental studies examined users’ perspectives on mobile apps and fitness trackers, reporting several barriers to their use, such as perceived lack of usefulness, loss of interest, and technical issues. The meta-analysis of RCTs showed a positive effect of mobile interventions on physical activity outcomes (standardized mean difference=0.45, 95% CI 0.17-0.73); several sensitivity analyses showed similar results. The trim-and-fill method showed possible publication bias. Only 20% (2/10) of the RCTs measured sedentary behaviors; both reported positive changes.

**Conclusions:**

The use of mobile interventions for physical activity and sedentary behaviors in the MENA region is in its early stages, with preliminary evidence of effectiveness. Policy makers and researchers should invest in high-quality studies to evaluate long-term effectiveness, intervention engagement, and implementation outcomes, which can inform the design of culturally and socially appropriate interventions for countries in the MENA region.

**Trial Registration:**

PROSPERO CRD42023392699; https://www.crd.york.ac.uk/prospero/display_record.php?RecordID=392699

## Introduction

### Background

Chronic diseases were responsible for >70% of deaths worldwide in 2019, making them the leading cause of mortality and morbidity [1]. In the Middle East and North Africa (MENA) region [2], chronic conditions accounted for 79% of deaths in 2020 [3]. Although there are many factors contributing to the prevalence of chronic diseases, physical inactivity and sedentary behaviors are well-established risk factors [4-8]. Worldwide studies have revealed that the MENA region has higher rates of physical inactivity compared with the global average, with 32.8% of adults and 85% of adolescents considered insufficiently active compared with 28% of adults and 81% of adolescents worldwide [9,10].

There are specific factors unique to the MENA region that might influence physical inactivity and sedentary behaviors, such as environmental conditions or infrastructure. Specifically, extreme weather conditions, particularly during the hot summer months, can make outdoor exercise uncomfortable or even hazardous [11,12]. Limited access to sports facilities is another barrier to physical activity, and high urbanization associated with dependence on motor vehicles likely increases sedentary time [11,12]. Given these regional factors, interventions targeting physical activity and sedentary behaviors in the MENA region need to be tailored to a regional and population context.

Mobile interventions such as mobile apps, fitness trackers, and SMS text messages can be a powerful tool for promoting physical activity and reducing sedentary behaviors. The high mobile penetration rate in the MENA region [13,14] makes mobile technologies a possible solution for delivering large-scale real-time interventions. Moreover, the advanced capability of these technologies to automate and process data can allow interventions to be tailored according to the specific individual, context, and region [15-18]. Mobile apps or fitness trackers can also incorporate theory-based behavior change techniques that are known to be effective [19], such as automating self-monitoring of activity and providing feedback or allowing users to set goals.

Despite this potential, to date, little is known about the effectiveness of mobile technologies targeting physical activity and sedentary behaviors in the MENA region. Although some systematic reviews have reported a positive effect of mobile technologies on behavioral outcomes [18,20-31], none of these studies have focused on evidence from the MENA region. A systematic review explored physical activity interventions in 6 Arabian Gulf countries (ie, Bahrain, Kuwait, Oman, Qatar, Saudi Arabia, and United Arab Emirates) [32]—a subset of the MENA region—but did not solely focus on mobile interventions or explore interventions for sedentariness. Thus, it remains unclear whether mobile technologies are particularly effective in changing physical activity and sedentary behaviors in the MENA region. In addition, given the unique barriers that the MENA population faces, there is also a need to understand users’ acceptability and the implementation outcomes of these mobile interventions (eg, the appropriateness or cultural fit of the interventions). Finally, it is worth noting that there is diversity among the countries in the MENA region regarding income level, economic and social stability [2], and mobile penetration rate [14]. Socioeconomic disparities have been linked to inequitable access to technologies and varying levels of digital literacy, creating a “digital divide” [33-35]. Understanding the geographical scope of mobile health research in the MENA region is crucial to gauge whether findings are applicable across the region and identify potential signs of a digital divide.

### Objectives

The aim of this systematic review and meta-analysis was to summarize the characteristics and evaluate the effectiveness of mobile interventions targeting physical activity and sedentary behaviors in the MENA region. A secondary aim was to explore users’ perspectives on these interventions as well as any other outcomes that might influence users’ adoption and use of mobile technologies (eg, appropriateness and cultural fit).

## Methods

This systematic review is reported in accordance with the PRISMA (Preferred Reporting Items for Systematic Reviews and Meta-Analyses) 2020 statement (Multimedia Appendix 1 [36]). We followed the protocol registered in PROSPERO (CRD42023392699).

### Search Strategy

A systematic search of the literature was conducted in MEDLINE, Embase, CINAHL, Scopus, and Global Index Medicus from database inception to January 19, 2023. The search strategy was developed after consultation with a research librarian and comprised three strings: (1) mobile technology terms, (2) physical activity and sedentary behavior terms, and (3) MENA countries (Multimedia Appendix 2). The reference lists of relevant articles and other reviews on similar topics were also screened to ensure that all eligible studies were captured. A gray literature search was performed using Google Scholar. We contacted the authors to obtain additional information when needed. We also contacted the authors to request the full text when it was not available. If the authors did not provide the full text, we excluded the studies.

### Eligibility Criteria

We included studies that met the following population, intervention, comparator, outcome, and study design criteria: (1) participants of all ages regardless of medical condition, (2) interventions that used mobile technologies (ie, mobile apps, fitness trackers, or SMS text messages), (3) presence or absence of a comparator, (4) any outcomes related to physical activity or sedentary behaviors (eg, active minutes and sitting time) or to users’ perceptions of mobile technologies, (5) conducted in MENA countries as listed by the World Bank [2], and (6) primary research studies (eg, randomized controlled trials [RCTs], quasi-experimental studies, or qualitative studies). Studies in all languages were included.

Studies were excluded if (1) the intervention did not have a mobile technology component (eg, web-based only) or (2) they only reported measures related to physical function (eg, sit-to-stand test). Multimedia Appendix 3 [2,20,37] provides a detailed outline of the eligibility criteria.

### Screening and Data Extraction

The screening procedure was piloted before beginning. In total, 4 pairs of investigators independently conducted a 2-phase screening using Rayyan (Rayyan Systems, Inc) [38] for title and abstract and full text. Disagreements were resolved through discussion.

The data extraction form was developed in Microsoft Excel (Microsoft Corp) and piloted before extraction. The following data were collected for each study: author; year; country; study design; study population, sample size, participant demographics, and baseline characteristics; details of the intervention and control conditions; retention rates (ie, percentage that completed the follow-up assessment); outcomes and times of measurement; and source of funding and conflicts of interest. Data were extracted by one researcher and checked for accuracy by another. RCTs were assessed by 2 independent researchers using the Cochrane risk-of-bias tool [39], and disagreements were resolved through discussion. To assess outcome reporting bias, we compared the outcomes specified in the trial protocols with the outcomes reported in the corresponding trial publications; if the trial protocols were unavailable, we compared the outcomes reported in the methods and results sections of the trial publications. Quality assessment of the rest of the included studies was completed using the Joanna Briggs Institute (JBI) critical appraisal tools, including the checklists for quasi-experimental studies, cross-sectional studies, and qualitative research [40].

### Strategies for Data Synthesis

A narrative synthesis was conducted of all included studies. A meta-analysis was conducted for RCTs in which the control group did not have a mobile technology component. This was because the study aimed to examine the effectiveness of mobile technologies on physical activity or sedentary behaviors compared with nonmobile interventions. Cluster randomized trials were also included in the meta-analysis by calculating the effective sample size using the Cochrane guideline [41] (Multimedia Appendix 4 [42,43]). We planned to perform 2 separate meta-analyses to combine outcome measures of physical activity and sedentary behaviors. However, only 2 trials measured sedentary behaviors; given this small number, a meta-analysis was used to combine physical activity outcomes only. Sedentary outcomes were summarized narratively.

Whenever a single study reported multiple outcomes for the same behavior (eg, reporting both daily step count and daily active minutes for physical activity), the outcome that was included in the meta-analysis was selected through the consensus of the authors favoring (1) the included studies’ primary outcomes, (2) the most meaningful outcomes to intended users (eg, step count), and (3) the longest follow-up. All reported outcomes (from data collected after the intervention) were pooled, and all effect sizes were transformed into standardized mean differences. We used a random-effects model for all analyses; the restricted maximum likelihood estimator was used to calculate the heterogeneity variance (τ^2^). To assess heterogeneity, *I*^2^ was used.

The presence and potential impact of publication bias were explored using a funnel plot, the Egger test, and the trim-and-fill method by Duval and Tweedie [44]. In total, 2 sensitivity analyses were conducted including only (1) studies that were randomized at the individual level and (2) studies that had a low risk of bias in at least 3 out of 5 categories. Owing to an insufficient number of studies, a planned meta-regression was not conducted. We used the Grading of Recommendations Assessment, Development, and Evaluation system for grading the body of evidence [45]. All computations were conducted in R (version 4.2.3; R Foundation for Statistical Computing) [46]. The 2-tailed significance level for all statistical tests was set at *P*<.05.

## Results

### Study Selection

**Figure 1:**
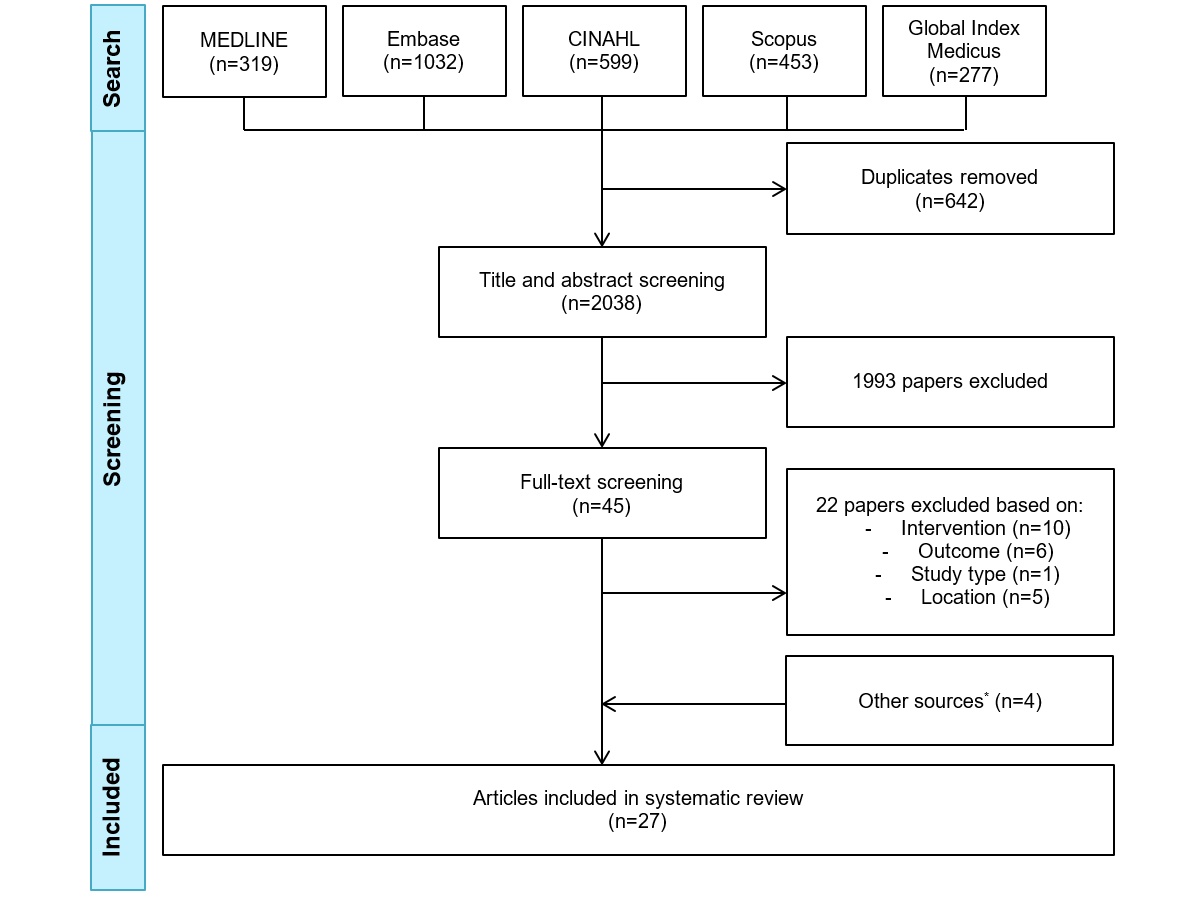
PRISMA (Preferred Reporting Items for Systematic Reviews and Meta-Analyses) flow diagram of the study selection process. *Other sources include reference lists of relevant articles (n=2) and Google Scholar (n=2).

The search retrieved 2038 unique articles (Figure 1). After abstract and full-text screening, 23 articles were included. Multimedia Appendix 5 provides a list of excluded studies at full-text screening. In total, 2 articles were found through the reference lists of relevant articles, and 2 were included from the Google Scholar search. Finally, the systematic review included 27 articles: 11 (41%) articles describing 10 unique RCT studies (n=2, 20% cluster RCTs [42,43,47]) [42,43,47-55], 12 (44%) quasi-experimental studies [56-67], 3 (11%) surveys [68-70], and 1 (4%) interview study [71] (Tables 1-3).

**Table 1:** Study and intervention characteristics of the included randomized controlled trials (RCTs).

Study, year, and country	Study population	Study length	Sample size, N	Female participants, n (%)	Age (y), mean	Details about the intervention	Details about the control
Ansari et al [55], 2022, Iran	Women aged 40-60 y	12 wk	IG^a^: 55; CG^b^: 55	110 (100)	52	The sample was divided into 2 groups: SMS text messaging or mobile social networking app; both received information about benefits and how to overcome potential barriers to physical activity.	No true control
Eslami et al [50], 2022, Iran	Pregnant women with overweight and obesity (ie, BMI >25 kg/m^2^)	8 wk	IG: 70; CG: 70	140 (100)	NR^c^; >55% in the 20-30–y age group	SMS text messages about physical activity (recommendations of amount and intensity, benefits, safe exercises, and precautions during pregnancy), diet, and supplements; educational booklet; and 1 face-to-face training session	Usual care
Saleh et al [48], 2022, Jordan	Patients with heart failure	8 wk	IG: 76; CG: 76	52 (39)	60.8	2 apps: Samsung Health app (goal setting and self-monitoring of activity) and social media app (posts about the benefits of physical activity, exercise videos, and discussion forum with a health expert; participants were encouraged to post and comment in the group)	Usual care; participants also received the setup for the Samsung Health app without receiving details about its features
Alshahrani et al [49], 2021, Saudi Arabia	Female university students	10 wk	IG: 53; CG: 50	103 (100)	NR; 70% were aged ≥20 y	WhatsApp group: a 15-min orientation and 3-4 messages/wk about physical activity	No intervention
Abbaspoor et al [51], 2020, Iran	Pregnant women with prediabetes	12 wk	IG: 50; CG: 50	100 (100)	IG: 27.8; CG: 29	2 SMS text messages every second day about gestational diabetes and 4 face-to-face training sessions	4 face-to-face training sessions
Alghafri et al [43,47], 2020 and 2018, Oman	Inactive adults with type 2 diabetes	1 y	Cluster RCT; IG: 122; CG: 110	137 (59)	44	Monthly WhatsApp messages, 3 face-to-face 20-min consultations about physical activity, and pedometers	Usual care
Parandeh et al [52], 2019, Iran	Women aged 30-45 y	8 wk	IG: 63; CG: 63	126 (100)	IG: 36.1; CG: 35.8	Daily SMS text messages about osteoporosis prevention (including physical activity and diet)	Educational SMS text messages about cancers in women
Quronfulah [42], 2019, Saudi Arabia	Male office workers	12 wk	Cluster RCT; IG: 33; CG: 33	0 (0)	43.5	Weekly SMS text messages targeting self-efficacy and self-regulation of physical activity, workplace computer prompts to take a break, videos of short bursts of exercise, and group and 1-1 educational sessions	No intervention
Alsaleh et al [53], 2016, Jordan	Outpatients with coronary heart disease	26 wk	IG: 71; CG: 85	72 (46)	IG: 57.7; CG: 58	SMS text messages (2/wk for the first 3 mo and 1/wk for the last 3 mo) with reminders to be active and meet personal goals; 1 face-to-face consultation to discuss barriers and facilitators and increase self-efficacy for physical activity; 6 phone calls to provide tailored feedback, review goals, and discuss any arising barriers; and paper diary to self-monitor physical activity	Usual care
Goodarzi et al [54], 2012, Iran	Adults with type 2 diabetes aged >30 y	12 wk	IG: 43; CG: 38	63 (77)	IG: 51; CG: 56.7	4 SMS text messages/wk about exercise, diet, diabetes medication, and importance of self-monitoring blood glucose levels	No intervention

^a^IG: intervention group.

^b^CG: control group.

^c^NR: not reported.

**Table 2:** Study and intervention characteristics of the included quasi-experiments.

Study, year, and country	Study population	Study length	Sample size, N	Female participants, n (%)	Age (y), mean	Details about the intervention and control (if applicable)	Physical activity and other outcomes
Al-Daghri et al [56], 2022, Saudi Arabia	Adolescents aged 12-16 y	12 mo	1 arm: 2600	343 (53)	14.8	Educational sessions (every 3 mo) about exercise or diet via Zoom or social media (WhatsApp, Telegram, Facebook, or Twitter), school-based educational sessions about type 2 diabetes and behavioral prevention, pamphlets, booklets, infographic videos, and gamification; participants with diabetes and prediabetes also received tailored advice via phone calls	NS^a^: physical activity Decrease in HbA_1c_^b^ in participants with diabetes and prediabetes (*P*<.001)
Ghofranipour et al [57], 2022, Iran	Mothers of children aged 3-5 y	6 wk	1 arm: 13	13 (100)	Mothers: 38; children: 4	WhatsApp messages and educational videos on reducing sedentary behaviors, increasing physical activity, and promoting a healthy diet	Increase in active minutes per day (*P*=.02) immediately and 3 mo after the intervention NS: BMI z score, diet, and quality of life
Ali et al [67], 2021, United Arab Emirates	Female university students with overweight or obesity aged 18-35 y	16 wk	2 arms—website: 54; website+app: 111	246 (100)	22	Website: self-monitoring of diet and physical activity, educational materials, web-based feedback and counseling, and news and weekly fitness challenges Website+app: self-monitoring of physical activity (PACER) and diet (MyNetDiary) via app, meeting with nutritionists, and WhatsApp messages	Website+app group: increase in number of days of moderate physical activity (*P*=.01) and minutes walked (*P*<.001), decrease in BMI (*P*=.04) and body fat (*P*<.001), and increase in nutritional knowledge (*P*<.001) NS in these domains in the website group Between-group significant decrease in waist circumference (*P*=.003) Of 11 mothers, 9 (81.8%) stated that the videos were practical, and 8 (72.7%) found them useful. Participants preferred the materials to be in Arabic, shorter, and delivered via mobile apps only.
Alyousef [58], 2021, Saudi Arabia	Women with type 2 diabetes	8 wk	1 arm: 20	20 (100)	54.8	WhatsApp messages with links to YouTube exercise videos and a phone call every 2 wk to encourage adherence	Increase in step count (*P*=.04) A total of 20 (100%) participants thought that the intervention was practical, they liked the videos, and they said that they would recommend it to family and friends. In total, 10% of the participants suggested a shorter video. Participants identified several barriers to following the YouTube exercises: lack of time, an appropriate place, and fast internet connection.
Biglar Chopoghlo et al [59], 2021, Iran	Female adolescents aged 14-18 y with type 1 diabetes	12 wk	2 arms—IG^c^: 38; CG^d^: 38	76 (100)	15.9	SMS text messages or MMS^e^ with educational contents regarding physical activity, diet, and other diabetes information CG: SMS text messages with nondiabetes content	NS in between-group exercises Between-group difference in self-efficacy (*P*<.001)
Khidir et al [60], 2021, Qatar	University staff and students	2 phases, 16 wk each	288 in phase 1 and 109 in phase 2	NR^f^; 56 women participated in both phases	NR	Mobile app: self-monitoring of physical activity and calories and fat burned Face-to-face presentation about benefits of physical activity, 4-mo walking competition, and pedometers	NS change in daily step count
Yahia and Bayoumi [61], 2021, Egypt	Outpatients with type 2 diabetes	12 wk	1 arm: 150	63 (42)	40.3	Mobile app (SOKARY): self-monitoring of physical activity, glucose level, and nutrition; medication reminders; and healthy lifestyle advice	Increased activity level (*P*<.001) Decreased HbA_1c_ (*P*=.03) and BMI (*P*=.006)
Jorvand et al [62], 2020, Iran	Health care workers	6 mo	2 arms—IG: 59; CG: 55	57 (50)	IG: 37.6; CG: 37.5	Mobile app (Telegram): educational content about physical activity and reminder messages to exercise; participants could also send pictures of their own exercise to the group CG: no intervention	Between-group difference in daily and weekly minutes of exercise (*P*<.001)
Alnasser et al [63], 2019, Saudi Arabia	Women with overweight or obesity	16 wk	1 arm: 240	240 (100)	31	Mobile app (Twazon): self-monitoring of step count and diet, recommendations for physical activity and diet, and setting of weight goals	NS: physical activity, weight, waist circumference, and BMI Usability score: within acceptable range
Lari et al [64], 2018, Iran	Outpatients with type 2 diabetes	12 wk	IG: 40; CG: 40	34 (47)	IG: 46.1; CG: 49.1	SMS text messages (2-3/d for 2 wk and then 2/wk) with recommendations for physical activity for diabetes and how to overcome barriers and seek social support CG: usual care	Between-group difference in MET^g^ min/wk (*P*<.001), perceived self-efficacy, barriers (*P*<.001), and family support (*P*=.046) NS: perceived health status, benefits, and friend support
Peyman et al [65], 2018, Iran	Women	6 mo	2 arms—IG: 180; CG: 180	360 (100)	IG: 33.4; CG: 31.9	Daily SMS text messages about the importance of physical activity; website with educational content and videos, assessment of physical activity and BMI, suggestions for women-only places for exercise, and chat room; and an educational CD CG: no intervention	Between-group difference in MET min/wk and knowledge and attitude about physical activity (*P*<.001)
Sani et al [66], 2018, Saudi Arabia	People who were diagnosed with type 2 diabetes within the last 5 y	6 mo	2 arms—IG: 100; CG: 100	100 (50)	NR; 75% were in the 30-49–y age group	SMS text messages and MMS in Arabic (2/wk) to encourage discussion; monthly meetings (input from specialist physicians, discussion, and peer group interactions); short presentations; video clips; and problem-based learning techniques about exercise, diet, and self-care CG: usual care	NS: physical activity, fasting blood glucose, diastolic blood pressure, triglycerides, and low-density lipoprotein Between-group difference in HbA_1c_ (*P*<.001), BMI, and systolic blood pressure (*P*<.001)

^a^NS: not significant.

^b^HbA_1c_: glycated hemoglobin.

^c^IG: intervention group.

^d^CG: control group.

^e^MMS: Multimedia Messaging Service.

^f^NR: not reported.

^g^MET: metabolic equivalent of task.

**Table 3:** Study information and summary of users’ perspectives and experiences in nonexperimental studies.

Study, year, and country	Study population	Study design	Sample size, N	Female participants, n (%)	Age (y), mean	Mobile technologies examined	Main findings
Al Ansari et al [68], 2023, Saudi Arabia	People aged >15 y	Survey	195	122 (63)	NR^a^; 40% were aged >40 y	Mobile apps	>50% agreed that the apps they used served all fitness levels. >80% agreed that it was easy to learn how to use the mobile apps. >70% agreed that mobile apps enhanced their knowledge of workouts and physical activity. NS^b^: between male and female participants and between people aged <40 y and people aged >40 y with respect to perceived usefulness and ease of use, attitudes, experiences, and subjective quality. Participants aged <40 y reported higher perceived ease of use than those aged >40 y.
Altabtabaei and Alhuwail [71], 2021, Kuwait	Students	Interviews	20	NR	NR	Fitness trackers	Participants’ main purposes of use were to lose weight and better understand and increase their physical activity levels. Barriers to adoption included perceived lack of usefulness, lack of knowledge about potential benefits and how to use fitness trackers, and concerns about battery life and data inaccuracy. Participants reported both positive (eg, more confidence) and negative (eg, guilt or stress when failing to achieve their activity goals) feelings associated with using fitness trackers.
Bardus et al [69], 2021, Lebanon	Student athletes	Survey	200	70 (35)	20	Mobile apps and fitness trackers	53% owned a fitness tracker; the main purpose of use was to facilitate self-monitoring. Reasons for discontinued use were loss of interest or technical issues.
Zaman et al [70], 2021, Saudi Arabia	People with sleep problems	Survey	45	23 (51)	NR; 51% were aged 20-30 y	Mobile apps	Participants reportedly improved their physical activity after using a health app (*P*=.009).

^a^NR: not reported.

^b^NS: not significant.

### Description of All Studies

Most of the included studies were conducted in Iran [50-52,54,55,57,59,62,64,65] (10/27, 37%) or Saudi Arabia [42,49,56,58,63,66,68,70] (8/27, 30%). In total, 7% (2/27) of the studies were conducted in Jordan [48,53] and Oman [43,47], and 4% (1/27) of the studies were conducted in Egypt [61], Kuwait [71], Lebanon [69], Qatar [60], and the United Arab Emirates each [67]. The included studies were published between 2012 and 2023. The average duration of experimental studies was 20 (SD 14.4; range 6-52) weeks. The total number of participants was 6141 (1/27, 4% of the studies had 2600 participants). Among the study participants, 46% were women; 7% (2/27) of the studies did not report gender distribution [60,71]. A total of 33% (9/27) of the studies were conducted in populations with chronic conditions, including cardiovascular diseases [48,53,61], diabetes [54,58,59,64,66], and sleep problems [70]. The funding sources and conflicts of interest are summarized in Multimedia Appendix 6 [42,43,47-71].

### Description of the Included Experimental Studies

#### Description of the Interventions

Half (11/22, 50%) of the interventions included a mobile app as a component [43,47-49,55-58,60-63,67]; 36% (4/11) of them used WhatsApp [43,47,49,57,58] (Tables 1 and 2). The other commercial apps used were Samsung Health [48] and Telegram [56,62]. In total, 19% (5/27) of the studies examined apps designed by the authors [55,60,61,63,67]. The main functions of mobile apps were to set goals, self-monitor activity, and receive educational information about physical activity [43,47-49,55-58,60-63,67]. Half (11/22, 50%) of the interventions used SMS text messaging to deliver educational content [42,50-55,59,64-66]; the frequency of delivery varied from 2 messages per day to 1 message per week.

In total, 36% (8/22) of the interventions also included a nonmobile component; the most popular were face-to-face sessions [42,43,47,50,51,53,60,66] (Tables 1 and 2). Other nonmobile components included websites [65,67], phone calls [53], computer prompts [42], pedometers [43,47,60], and an educational booklet and CD [50,65]. In total, 37% (10/27) of the studies mentioned that the interventions were designed using behavior change theories [42,43,48,51,53,57,58,63,64,67], the most popular being social cognitive theory (4/10, 40%) [42,53,63,67]. Self-efficacy constructs [53,57] and the health promotion model [58,64] were used in 20% (2/10) of the studies each.

#### Description of the Control Groups

Of the 10 included RCTs, 3 (30%) had a true control group (ie, no intervention) [42,49,54] (Table 1). A total of 30% (3/10) had an active mobile control [48,52,55]. Specifically, in 10% (1/10) of the studies, the control group received usual care (ie, clinical consultation) as well as the Samsung Health app without being told about its features [48]; the intervention was guided by the theory of planned behavior. In another study, the intervention group received daily SMS text messages about physical activity, whereas the control group received educational SMS text messages about cancers; no theory was mentioned [52]. In the third study, the content of the intervention and comparator arms used the same behavior change techniques (ie, information about health consequences), the only difference being the delivery platform (ie, mobile app vs SMS text messaging); no theory was mentioned [55]. A total of 40% (4/10) of the RCTs had nonmobile controls [43,47,50,51,53]; all mentioned the use of a theory. In total, 75% (3/4) of these RCTs involved usual care in the control arm [43,47,50,53], and 25% (1/4) involved face-to-face education [51].

A total of 50% (6/12) of the quasi-experiments also had a control arm, of which 33% (2/6) were true controls (ie, no intervention [62,65]) and 33% (2/6) were usual care [64,66]. A total of 33% (2/6) of the studies involved an active digital control. In one study, the control group received SMS text messages that were not related to physical activity [59]; no theory was mentioned. In another study, the control group had access to a website with the self-monitoring, receiving feedback, goal setting, and information about health consequences behavior change techniques [67]; social cognitive theory was used to develop the intervention.

#### Engagement and Retention Metrics

None of the included RCTs reported metrics of engagement with the interventions. A total of 25% (3/12) of the quasi-experiments reported engagement metrics [57,60,63]. Specifically, one study reported that 23% (3/13) of mothers watched all videos delivered via WhatsApp (Multimedia Appendix 6) [57]. Another study reported a higher use of mobile apps than pedometers to monitor activity [60]. One study measured use rates and reported that, at 6 months, 55% (26/47) of the participants used the app at least once every 2 weeks [63].

The retention rate in the intervention groups ranged from 18% to 100%. The average retention rate for the intervention arms was 85%, with most falling within the 86% to 100% range. A total of 22% (6/27) of the studies had a 100% retention rate [50,55,57,61,62,65], and 7% (2/27) had a retention rate of <25% [56,63] (Multimedia Appendix 6).

#### Users’ Perspectives in Experimental Studies

Of the 27 studies, 4 (15%) experimental studies (n=3, 75% RCTs [42,47,53] and n=1, 25% quasi-experiments [58]) examined users’ perspectives and experiences with the mobile interventions (Table 2 and Multimedia Appendix 7 [42,43,47-67]). Most of the participants reported finding the interventions useful; some reported benefits such as gaining knowledge about how to change their behaviors, receiving reminders to be more active, and building a relationship with clinicians [53]. One study reported barriers to using the mobile intervention such as lack of reliable internet connection and lack of time and an appropriate place to exercise [58]. In one study, participants mentioned a preference for the intervention material to be linguistically adapted [67].

#### Users’ Perspectives in Nonexperimental Studies

Of the 4 nonexperimental studies, 2 (50%) examined the role of mobile apps [68,70], 1 (25%) focused on fitness trackers [71], and 1 (25%) investigated both [69] (Table 3). A total of 50% (2/4) of the studies found that the main purpose of use was to self-monitor their activity [69,71]. Barriers to adoption and use included perceived lack of usefulness, lack of knowledge about potential benefits, loss of interest, and technical issues [69,71]. One survey found that people aged <40 years reported higher perceived ease of use than those aged >40 years [68]. A survey of people with sleep problems reported improved physical activity after using a health app [70].

### Quality Assessment

The risk of bias was assessed as low in 3 out of 5 categories in half (5/10, 50%) of the included RCTs [43,48,50,53,55] (Table 4). Half (5/10, 50%) of the included RCTs described a low-risk randomization process [48,51-53,55]. In 20% (2/10) of the trials, the allocation sequence was not concealed until participants were enrolled [42,49]. Of the 10 RCTs, 2 (20%) cluster RCTs were assessed as low risk in an additional domain (ie, “timing of identification or recruitment of participants”). The risk of “deviations from intended interventions” was assessed as low in 40% (4/10) of the studies [43,50,53,55], there were “some concerns” in 40% (4/10) of the studies [42,48,51,52], and the risk was high in 20% (2/10) of the studies as an appropriate analysis (eg, intention-to-treat) was not used. Half (5/10, 50%) of the included RCTs had a low level of incomplete data [42,43,47,48,55], whereas the other half were assessed as having some concerns [49,51-54]. Most of the studies (7/10, 70%) were assessed as having some concerns regarding the measurement of the outcomes because of the self-report nature, and participants (ie, outcome assessors in this case) were aware of the intervention allocation [43,49-51,53-55]. More than half (6/10, 60%) of the included studies scored as having a low risk in the selection of the reported results [43,49-51,53,55], whereas 40% (4/10) of the studies were assessed as having some concerns because of the lack of details regarding a preplanned analysis [42,48,52,54].

**Table 4:** Risk-of-bias assessment of the included randomized controlled trials. Green: low risk; yellow: some concerns; red: high risk.

Study, year	Randomization process	Deviations from intended interventions	Missing outcome data	Measurement of the outcome	Selection of the reported result	Overall
Abbaspoor et al [51], 2020						
Alghafri et al [43], 2018						
Alsaleh et al [53], 2016						
Alshahrani et al [49], 2021	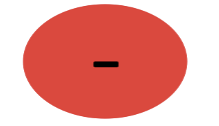	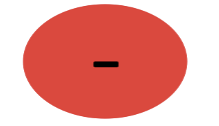				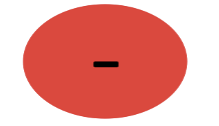
Ansari et al [55], 2022						
Eslami et al [50], 2022						
Goodarzi et al [54], 2012		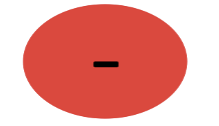				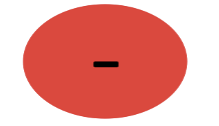
Parandeh et al [52], 2019						
Quronfulah [42], 2019	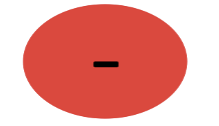					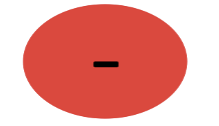
Saleh et al [48], 2022						

Table 5 provides a summary of the quality assessment of the quasi-experimental studies using the JBI checklist for quasi-experiments. All 12 quasi-experimental studies [56-67] made clear what was the cause versus the effect (question 1), had multiple measurements of the outcome both before and after the intervention (question 5), and measured the outcomes of participants in the comparison groups in the same way as those of participants in the intervention groups (question 7). In all except one study (11/12, 92%) where this was unclear [67], participants in the comparison group were similar to participants in the intervention group. In one study [65], it was unclear whether participants in the comparison group received similar treatment; the other 92% (11/12) of the studies met this criterion (question 3). In half (6/12, 50%) of the studies, there was no control group (question 4). Only 8% (1/12) of the studies provided information about whether follow-up was complete and how follow-up data were treated [62] (question 6). In total, 83% (10/12) of the studies measured outcomes in a reliable way; this information was unclear in 17% (2/12) of the studies [57,61] (question 8). Appropriate statistical analyses were conducted in 92% (11/12) of the studies; this was unclear in 8% (1/12) of the studies [61] (question 9).

The survey studies (3/27, 11%) [68-70] were assessed using the JBI checklist for cross-sectional studies (Table 6). The criteria for inclusion were not clearly defined in those 3 studies (question 1). The study participants and settings were described in detail in 67% (2/3) of the studies [68,69] (question 2). Owing to poor reporting, it was unclear in all 3 studies whether exposure and outcomes were measured in a valid and reliable way (questions 3 and 7) and whether confounding factors and strategies to deal with them were identified (questions 5 and 6). In 33% (1/3) of the studies, appropriate statistical analysis was used (question 9).

**Table 5:** Quality assessment of the included quasi-experimental studies.

Study, year	Q1^a^	Q2	Q3	Q4	Q5	Q6	Q7	Q8	Q9
Al-Daghri et al [56], 2022	Y^b^	Y	Y	N^c^	Y	U^d^	Y	Y	Y
Ghofranipour et al [57], 2022	Y	Y	Y	N	Y	U	Y	U	Y
Ali et al [67], 2021	Y	U	Y	Y	Y	N	Y	Y	Y
Alyousef [58], 2021	Y	Y	Y	N	Y	U	Y	Y	Y
Biglar Chopoghlo et al [59], 2021	Y	Y	Y	Y	Y	N	Y	Y	Y
Khidir et al [60], 2021	Y	Y	Y	N	Y	U	Y	Y	Y
Yahia and Bayoumi [61], 2021	Y	Y	Y	N	Y	U	Y	U	U
Jorvand et al [62], 2020	Y	Y	Y	Y	Y	Y	Y	Y	Y
Alnasser et al [63], 2019	Y	Y	Y	N	Y	N	Y	Y	Y
Lari et al [64], 2018	Y	Y	Y	Y	Y	N	Y	Y	Y
Peyman et al [65], 2018	Y	Y	U	Y	Y	U	Y	Y	Y
Sani et al [66], 2018	Y	Y	Y	Y	Y	N	Y	Y	Y

^a^Q: question.

^b^Y: yes.

^c^N: no.

^d^U: unclear.

**Table 6:** Quality assessment of the included survey studies.

Study, year	Q1^a^	Q2	Q3	Q4	Q5	Q6	Q7	Q8
Al Ansari et al [68], 2023	N^b^	Y^c^	U^d^	N/A^e^	U	U	U	U
Bardus et al [69], 2021	N	Y	U	N/A	U	U	U	Y
Zaman et al [70], 2021	N	N	U	N/A	U	U	U	U

^a^Q: question.

^b^N: no.

^c^Y: yes.

^d^U: unclear.

^e^N/A: not applicable.

The interview study [71] was assessed using the JBI checklist for qualitative research. It was assessed as meeting the criteria in 5 domains (ie, congruity between the research methodology and the research question, congruity between the research methodology and the methods to collect data, congruity between the research methodology and the representation and analysis of data, representation of participants and their voices, and ethics approval by an appropriate body). In total, 3 domains were unclear (ie, congruity between the stated philosophical perspective and the research methodology, congruity between the research methodology and the interpretation of results, and the relationship between the conclusions and the analysis or interpretation of the data). There was no statement locating the researcher culturally or theoretically or on the influence of the researcher on the study.

### Meta-Analysis of RCTs and Outcomes in Quasi-Experiments

Of the 10 included RCTs, 7 (70%) were deemed eligible for inclusion in the meta-analysis of physical activity outcomes. Specifically, 20% (2/10) of the RCTs were excluded because they had a mobile component in the control arm [48,55], and 10% (1/10) were excluded for only reporting behavioral practices regarding diabetes care (combining physical activity and other health measures) [54]. In line with the strategies for data synthesis, for 71% (5/7) of the studies [43,49-52], the measures of metabolic equivalent of task minutes per week were included in the meta-analysis; for the other 29% (2/7) of the studies, the duration of moderate to vigorous physical activity was included [42,53].

**Figure 2:**
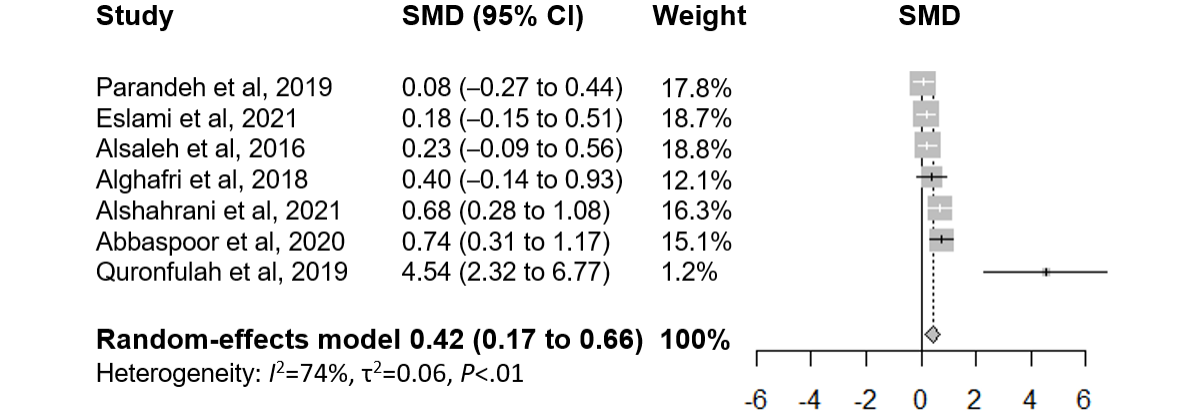
Forest plot of effect sizes and 95% CIs of physical activity outcomes ordered by descending effect size of the individual studies. SMD: standardized mean difference.

The meta-analysis showed a positive effect of mobile interventions on physical activity outcomes (standardized mean difference=0.45, 95% CI 0.17-0.73; Figure 2 [42,43,49-53]). The *I*^2^ was 74%, indicating a high heterogeneity. One study in particular [42] seemed to be the source of high heterogeneity. This was the only study that delivered an intervention in the workplace, with part of the intervention including regular computer prompts throughout the day. The high frequency of the computer prompts might have had an effect on behavior changes. The funnel plot appeared to indicate signs of publication bias (Multimedia Appendix 8). The Egger test had an intercept of 3.65 (*P*=.02), indicating possible publication bias. The trim-and-fill method imputed an effect size of 0.30 (95% CI −0.65 to 1.25), indicating a possible nonsignificant effect. In total, 3 sensitivity analyses were conducted, including only (1) studies that were randomized at the individual level and (2) studies that had a low risk of bias in at least 3 categories and excluding the study by Quronfulah [42] as an outlier. All 3 sensitivity analyses showed a statistically significant positive result for physical activity (*P*<.05; Multimedia Appendix 9). Subgroup analyses were conducted among studies with patients who were chronically ill and studies with healthy individuals; both reported nonsignificant changes in physical activity (Multimedia Appendix 9). Only 20% (2/10) of the RCTs reported measures regarding sedentary behaviors; both reported a greater reduction in sedentary time in the intervention group than in the control group [42,43] (Multimedia Appendix 7).

Quasi-experiments were not included in the meta-analysis because of the generally lower quality and lack of a control group. Over half (7/12, 58%) of the quasi-experiments reported significant changes in physical activity [57,58,61,62,64,65,67]. Other anthropometric (eg, BMI and weight) or clinical (eg, glycated hemoglobin) outcomes were also reported (Table 2). It is worth noting that most experimental studies (19/22, 86%) measured physical activity outcomes using a validated questionnaire (eg, the International Physical Activity Questionnaire); only 20% (2/10) of the RCTs [42,43] and 8% (1/12) of the quasi-experiments [48] (3/27, 11% in total) used an objective method (eg, accelerometer).

## Discussion

### Principal Findings

Our review revealed that the use of mobile technologies for physical activity and sedentary behaviors in the MENA region is at an early stage of research given the predominance of small and heterogeneous studies and very few RCTs. The meta-analysis provided preliminary evidence of a small to moderate positive effect of mobile interventions on physical activity in the short term; only 7% (2/27) of the studies measured sedentary behaviors, reporting positive outcomes from the interventions. It is important to note that this evidence is of low to moderate quality according to the Grading of Recommendations Assessment, Development, and Evaluation system [45] because of the high risk of bias in half (5/10, 50%) of the assessed RCTs, heterogeneity in intervention delivery and context, short study duration, possible small-study effects, and publication bias. The reporting standards of the included studies varied widely, and only a small number of studies (3/22, 14%) provided metrics of engagement with the mobile interventions. Nonexperimental studies revealed that users mainly used mobile apps and fitness trackers for self-monitoring of physical activity. Users also revealed several barriers to technology adoption; few studies (1/27, 4%) evaluated the implementation outcomes (eg, context-specific or cultural appropriateness) of the interventions.

### Comparison With Existing Literature

This systematic review and meta-analysis focused on the MENA region and provided insights into the effectiveness of mobile technologies on physical activity and sedentary behaviors. Similar to the findings of our meta-analysis, a number of systematic reviews have found a small to moderate effect of mobile technologies on behavioral outcomes [18,20-31]; however, these reviews did not focus on a specific region, and thus, evidence applicable to the MENA region was limited. Furthermore, as these reviews were not MENA specific, it is unclear what would be the most promising interventions given the unique barriers that the MENA region faces. Our review found that particularly favorable interventions were those that conducted user needs assessments, considered daily lives and cultural contexts, and had multicomponent approaches. These findings are in line with those of a review focused on the Asian population that found that Asian apps are largely culturally adapted and multifunctional [72]. A few reviews have explored the use of technologies in specific regions, such as low- and middle-income countries or Asia [72,73], which also showed promising evidence of using these technologies for physical activity and other behavior changes. One systematic review explored physical activity interventions in only 6 Arabian Gulf countries [32] and did not exclusively examine mobile interventions or explore interventions for sedentariness. This review found limited evidence suggesting that pedometer-based interventions encouraging step counting and walking were effective in promoting physical activity, which might suggest the potential of using mobile technologies that can automate self-monitoring of behavior [32]. Thus, our review expands on existing evidence by investigating the use of mobile interventions in MENA countries for both physical activity and sedentary behaviors and, hence, is able to assess whether mobile technologies were effective for this population and what the users’ perspectives were. Combined with the findings from previous reviews, there is evidence suggesting that mobile technologies might be helpful in changing physical activity; however, these interventions likely need to be tailored to users’ daily lives and cultural contexts.

Our meta-analysis suggests that mobile technologies may be promising for changing physical activity and sedentary behavior in the MENA region. Particularly favorable interventions were those that conducted user needs assessments, considered daily lives and cultural contexts, and had multicomponent approaches. For example, one study assessed users’ preferred method of communication and intervention frequency to tailor the design accordingly, resulting in a high level of acceptability and perceived usefulness [42]. Interestingly, WhatsApp was identified as a preferred platform and used in several studies to deliver educational content or send reminders [43,47,49,57,58]. Conducting needs assessments during intervention development will allow researchers and policy makers to determine whether existing technologies can be leveraged or whether additional apps or features are required to meet users’ needs. Incorporating cultural contexts, such as scheduling intervention messages during cultural or health events (eg, Ramadhan and World Hypertension Day) [43], was also a promising approach. In addition, successful interventions combined mobile technologies with face-to-face consultations, suggesting that mobile technologies can complement the role of periodic in-person consultations by delivering more frequent support in daily contexts. It is worth noting that, in the meta-analysis, studies that had a true control group also tended to report larger effect sizes and significant results. Overall, our findings emphasize the potential of mobile technologies to promote behavior changes in the MENA region, necessitating strategies that consider user needs, cultural fit, and multicomponent approaches.

Our review revealed several gaps involving country-specific contexts, targeted populations, and behaviors that should be addressed by researchers and policy makers. First, two-thirds of the included studies (18/27, 67%) were conducted in Saudi Arabia and Iran, and thus, evidence on the use of mobile technologies in other MENA countries was limited. It is likely that more research has been conducted in stable and high-income countries given the diversity of countries in the MENA region regarding income level, economic and social stability [2], and mobile penetration rate [14]. This finding flags the issues of equitable access to mobile technologies across the region, potentially worsening the digital divide and widening health gaps [33-35]. The benefits of mobile health will be limited if it can only reach people with a high socioeconomic status. Thus, concentrated efforts are essential to increase technology access across the region and promote broader research initiatives across countries and contexts.

Second, evidence on mobile interventions for children and adolescents remains limited, with only one included intervention targeting mothers with the ultimate aim of changing preschoolers’ physical activity and diet. Given the importance of promoting healthy behaviors from an early age, policy makers should invest in the development and evaluation of mobile interventions for children and adolescents in the MENA region. Third, only 7% (2/27) of the studies intervened on and measured sedentary behaviors, highlighting the need for future research and investment from policy makers to address sedentariness so as to holistically address inactive lifestyles.

In addition, none of the experimental studies examined fitness trackers as part of the intervention despite interest in these devices (as reported in 2/4, 50% of the nonexperimental studies [69,71]) and existing evidence of their positive effects on physical activity [18,20,22,23,25]. Finally, most RCTs (7/10, 70%) measured behavioral outcomes using self-reporting methods (eg, validated questionnaires). Future research might consider using objective measures provided by mobile apps and fitness trackers, which can improve the accuracy and reliability of the data and provide more robust evidence for policy decision-making.

The reporting standards of the included studies in our review varied greatly, with few details provided regarding the intervention, study procedure, and methodology in some studies. Notably, few of the included experimental studies (3/22, 14%) assessed user engagement even though a large body of research has suggested that engagement with digital interventions is a precondition for effectiveness and highlighted issues of high dropout or nonuse attrition in mobile interventions. Future studies should adhere to reporting guidelines (eg, CONSORT [Consolidated Standards of Reporting Trials], STROBE [Strengthening the Reporting of Observational Studies in Epidemiology], and COREQ [Consolidated Criteria for Reporting Qualitative Research]) [74-76] to enable evidence synthesis and assess engagement metrics consistently to allow for future evaluation of the right “dose” of use of these mobile technologies for effectiveness.

Finally, our review also identified qualitative evidence on barriers to adoption and user preferences that policy makers and researchers should consider. Factors such as perceived lack of usefulness, loss of interest, and technical issues can hinder the effectiveness of mobile interventions [77,78]. In addition, although some participants mentioned a preference for intervention material being culturally and linguistically adapted, few studies (1/27, 4%) examined the context fit or cultural appropriateness of the interventions, highlighting the need for future evaluation of these implementation outcomes.

### Strengths and Limitations

Our study has several strengths. We followed a prespecified protocol registered in the PROSPERO database. Our search included peer-reviewed and gray literature. We also hand searched related reviews to ensure that relevant studies were captured. Data extraction and risk-of-bias assessment were conducted by 2 reviewers, and the authors were contacted for additional information. Finally, we conducted several sensitivity analyses (which were consistent with our main results) and assessed risk of bias and publication bias to better understand the limitations of our findings.

Our findings should be interpreted within the context of the study’s limitations. The poor reporting of the included studies affected our synthesis capability. Owing to the small number of studies targeting sedentary behaviors, it was not possible to conduct a meta-analysis of this behavioral outcome. The meta-analysis findings were affected by the quality of the included studies, including a large proportion of self-reported outcomes, small sample sizes, and possible publication bias. Our data synthesis strategy selected one outcome from each study to be included in the meta-analysis; future research might consider performing a multilevel meta-analysis to include all reported outcomes of the studies [79]. In addition, as most of the included studies (18/27, 67%) were conducted in Saudi Arabia or Iran, it is important to acknowledge the potential limitations in generalizing the findings to other countries in the MENA region. However, the insights derived from this review can still serve as a valuable guide for future research endeavors and inform policy-making processes in other MENA countries.

### Implications

Our findings have important implications for policy, practice, and future research in the MENA region. First, regarding policy implications, policy makers should support and fund RCTs with longer durations to determine the long-term effectiveness of mobile technologies on physical activity and sedentary behavior. Investment should also be made in technology infrastructure and research initiatives in countries with lower socioeconomic status to promote equitable access to mobile technologies across the MENA region. In addition, policy makers need to ensure that interventions are culturally sensitive and linguistically adapted to enhance their acceptability and effectiveness. Finally, it is important to recognize that mobile technologies alone are unlikely to address behavioral challenges in the MENA region given the variability in mobile ownership and social and economic conditions [2,14,80,81]. Thus, policy makers will need to direct efforts into designing and evaluating multifaceted interventions that can appropriately target physical activity and sedentary behaviors while considering the diverse country contexts.

The evidence of the preliminary effectiveness of mobile interventions found in this review needs to be supported by future rigorous evaluations, with the ultimate goal of assisting clinical practice. If there is sufficient, high-quality evidence, clinicians may consider discussing the use of mobile interventions with people who need to change their activity levels as part of a shared decision-making process [82,83]. Potentially, mobile technologies can enhance patient-clinician collaboration by capturing data to facilitate period review while also empowering individuals to manage their health more actively [84]. As research in the MENA region continues to evolve, evidence of the effectiveness of mobile technologies can be used to determine whether their use can become part of routine clinical care. It is important to note that, although technology prescription is a promising prospect, clinicians have reported several barriers to this practice—the most prominent concern being the lack of knowledge of prescribable technologies and lack of reliable sources to access this information [85,86]. Therefore, a nationally accessible repository of vetted and curated technologies for health care professionals is needed to promote the sustainability and scalability of mobile technologies in clinical practice [85,86].

Implications for future research encompass the need for well-designed RCTs, adherence to reporting standards, and assessment of implementation outcomes. First, well-designed and fully powered RCTs are needed to provide high-quality evidence on the effectiveness of mobile technologies on physical activity and sedentary behaviors, especially over a long-term follow-up. Second, it is crucial for future studies to adhere to existing reporting guidelines [74,75,87] to facilitate evidence synthesis on the most effective intervention, “dosage,” or delivery channel. Finally, researchers should consistently assess and report intervention engagement, acceptability, and implementation outcomes (eg, cultural fit, sustainability, and cost-effectiveness) [76] to determine the viability of mobile technologies for behavior change and successful implementation in the MENA context.

### Conclusions

Our systematic review and meta-analysis found that research in the MENA region on the use of mobile interventions for physical activity and sedentary behaviors is in its early stages, with preliminary evidence indicating their effectiveness. However, the studies varied greatly in terms of intervention components, methodology, and study quality, and therefore, the findings must be interpreted with caution. Policy makers and researchers need to invest in high-quality studies to evaluate the effectiveness, engagement, and implementation process of mobile interventions in the MENA region.

## Appendix

Multimedia Appendix 1 PRISMA (Preferred Reporting Items for Systematic Reviews and Meta-Analyses) statement.

Multimedia Appendix 2 Search strategy.

Multimedia Appendix 3 Eligibility criteria.

Multimedia Appendix 4 Calculating the effective sample size of cluster randomized controlled trials for the meta-analysis using Cochrane guidelines.

Multimedia Appendix 5 List of excluded studies after full-text review for not meeting the inclusion criteria regarding intervention or outcome.

Multimedia Appendix 6 Information about funding sources and conflicts of interest of the included studies.

Multimedia Appendix 7 Retention rates, engagement metrics, and other outcomes from the experimental studies.

Multimedia Appendix 8 Funnel plot of SE by standardized mean difference.

Multimedia Appendix 9 Sensitivity and subgroup analyses.

## Abbreviations

CONSORT: Consolidated Standards of Reporting Trials
COREQ: Consolidated Criteria for Reporting Qualitative Research
JBI: Joanna Briggs Institute
MENA: Middle East and North Africa
PRISMA: Preferred Reporting Items for Systematic Reviews and Meta-Analyses
RCT: randomized controlled trial
STROBE: Strengthening the Reporting of Observational Studies in Epidemiology

## Data Availability

The data sets generated during and analyzed during this study are available from the corresponding author on reasonable request.
