# Supplementary material for: The Use of Mobile Technologies to Promote Physical Activity and Reduce Sedentary Behaviors in the Middle East and North Africa Region: Systematic Review and Meta-Analysis
Source: J Med Internet Res. 2024 Mar 19;26:e53651. doi: 10.2196/53651 (PMC10988381; doi:10.2196/53651)
Supplement: Multimedia Appendix 2 [file jmir_v26i1e53651_app2.docx]

# Appendix 2: Search strategy

Note:

- For all databases, the search was conducted on 19 January 2023.

1. **Search strategy for MEDLINE (via OVID interface)**

#1: physical activit*.mp. or exp Exercise/ or Physical Fitness/ or fitness.mp. or Sedentary Behavior/ or sedentar*.mp. or walk*.mp. or Walking/ or Running/ or run*.mp. or sport*.mp. or exp Sports/ or Jogging/ or jog*.mp. or Bicycling/ or (biking or bicycling).mp. or swim*.mp. or Swimming/ or cool-down exercise/ or gymnastics/ or muscle stretching exercises/ or circuit-based exercise/ or endurance training/ or high-intensity interval training/ or plyometric exercise/ or resistance training/ or warm-up exercise/ or Weight Lifting/ or weight lift*.mp. or strength train*.mp. or resistance train*.mp. or circuit train*.mp. or circuit exercise*.mp. or weight train*.mp. or aerobic train*.mp. or aerobic exercise*.mp. or cardio train*.mp. or HIIT.mp. or high intensity interval train*.mp. or step count*.mp. or Screen Time/ or screen behavio*.mp. or screen time.mp. or Television/ or screen watching.mp. or sitting time.mp. or television.mp. or TV time.mp. or TV viewing.mp. or TV watching.mp. or watch TV.mp. or watch television.mp. or view television.mp. or prolonged sitting.mp. or lying time.mp. or physical inactiv*.mp. or screen entertainment.mp. or screen-based entertainment.mp. or video gam*.mp. or Video Games/ or computer gam*.mp.

AND

#2: mobile app*.mp. or Mobile Applications/ or mobile health.mp. or Telemedicine/ or (mhealth or m-health).mp. or Smartphone/ or mobile technolog*.mp. or Text Messaging/ or smartphone*.mp. or fitness tracker*.mp. or Fitness Trackers/ or wearable device*.mp. or Wearable Electronic Devices/ or wearable technolog*.mp. or smartwatch*.mp. or smart watch*.mp. or activity tracker*.mp. or short messag* service*.mp. or (text messag* or texting) or MMS.mp. or multimedia messag* service*.mp. or whatsapp or social media.mp. or Social Media/ or Cell Phone/ or cellphone*.mp. or cell phone*.mp. or Accelerometry/ or accelerometer*.mp. or ehealth.mp. or e-health.mp. or Internet/

AND

#3: middle east*.mp. or exp Middle East/ or islam*.mp. or exp Islam/ or arab*.mp. or exp Arabs/ or arab world.mp. or exp Arab World/ or exp africa, northern/ or north africa*.mp. or north-africa*.mp. or algeria*.mp. or exp Algeria/ or bahrain*.mp. or exp Bahrain/ or djibouti*.mp. or exp Djibouti/ or exp Egypt/ or egypt*.mp. or jordan*.mp. or exp Jordan/ or kuwait*.mp. or exp Kuwait/ or lebanon*.mp. or exp Lebanon/ or libya*.mp. or exp Libya/ or iran*.mp. or exp Iran/ or exp Iraq/ or iraq*.mp. or israel*.mp. or exp Israel/ or (malta* or maltese).mp. or exp Malta/ or morocc*.mp. or exp Morocco/ or oman*.mp. or exp Oman/ or qatar*.mp. or exp Qatar/ or saudi arabia*.mp. or exp Saudi Arabia/ or syria*.mp. or exp Syria/ or tunisia*.mp. or exp Tunisia/ or (united arab emirates or emirati* or UAE).mp. or exp United Arab Emirates/ or west bank.mp. or gaza*.mp. or yemen*.mp. or exp Yemen/ or palestin*.mp. or gulf.mp. or africa/ or Mediterranean Region/ or eastern mediterranean.mp. or hebrew.mp.

1. **Search strategy for Embase (via OVID interface)**

#1: exp physical activity/ or exp exercise/ or physical activit*.mp. or physical fitness.mp. or fitness/ or sedentary time/ or sedentary lifestyle/ or sedentary.mp. or sedentar*.mp. or walk*.mp. or walking/ or run*.mp. or running/ or contact sport/ or aeronautical sport/ or endurance sport/ or team sport/ or sport*.mp. or sport/ or jogging/ or jog*.mp. or bicycling.mp. or cycling/ or biking.mp. or swimming/ or swim.mp. or gymnastic*.mp. or gymnastics/ or weight lifting/ or weight lift*.mp. or resistance training/ or strength train*.mp. or resistance train*.mp. or circuit training/ or circuit train*.mp. or circuit exercise*.mp. or weight train*.mp. or weight training/ or aerobic train*.mp. or cardio* train*.mp. or physical train*.mp. or power training/ or endurance training/ or resistance training/ or sprint interval training/ or interval training/ or "cross training (sport)"/ or high intensity interval training/ or moderate intensity continuous training/ or high intensity interval train*.mp. or HIIT.mp. or muscle exercise/ or muscle exercise*.mp. or muscle stretching/ or stretch* exercise*.mp. or stretching exercise/ or step count/ or step count*.mp. or weight bearing/ or weight bear*.mp. or stretching/ or stretching.mp. or open kinetic chain exercise/ or aquatic exercise/ or leg exercise/ or anaerobic exercise/ or aerobic exercise/ or closed kinetic chain exercise/ or high intensity exercise/ or isotonic exercise/ or moderate intensity exercise/ or arm exercise/ or isokinetic exercise/ or isometric exercise/ or treadmill exercise/ or "squatting (exercise)"/ or low intensity exercise/ or static exercise/ or dynamic exercise/ or exercise*.mp. or continuous training/ or cool down/ or pilates/ or pilate*.mp. or plyometrics/ or jumping/ or warm up/ or screen time.mp. or screen time/ or television viewing/ or binge watching/ or binge watch*.mp. or screen behavio*.mp. or screen watch*.mp. or sitting/ or sit* time.mp. or television.mp. or television/ or TV time.mp. or TV view*.mp. or TV watch*.mp. or watch TV.mp. or watch television.mp. or view television.mp. or prolonged sit*.mp. or lying time.mp. or physical inactivity/ or physical inactiv*.mp. or screen entertainment.mp. or screen-based entertainment.mp. or video game/ or video gam*.mp. or computer gam*.mp. or computer game/

AND

#2: mobile application/ or mobile phone/ or mobile app*.mp. or telemedicine/ or telehealth/ or mobile health.mp. or mobile health application/ or mhealth.mp. or m-health.mp. or smartphone/ or smartphone*.mp. or mobile technolog*.mp. or text messaging/ or text messag*.mp. or Internet/ or short messag* service*.mp. or texting.mp. or MMS.mp. or multimedia messag* service*.mp. or activity tracker/ or fitness tracker*.mp. or wearable device*.mp. or wearable computer/ or wearable sensor/ or smart watch/ or activity tracker/ or wearable technolog*.mp. or smartwatch*.mp. or smart watch*.mp. or activity tracker*.mp. or whatsapp.mp. or social media/ or social media.mp. or "cell phone use"/ or cellphone*.mp. or mobile phone/ or cell phone*.mp. or accelerometer/ or accelerometry/ or accelerometer*.mp. or ehealth.mp. or e-health.mp.

AND

#3: exp Middle East/ or middle east*.mp. or exp Islam/ or islam*.mp. or bedouin/ or exp arab/ or exp arab world/ or arab*.mp. or arab world.mp. or exp north african/ or exp North Africa/ or north* africa*.mp. or Africa/ or north-africa*.mp. or exp Algeria/ or algeria*.mp. or exp Algerian/ or exp Bahrain/ or bahrain*.mp. or bahraini.mp. or exp Bahraini/ or exp Djibouti/ or djibouti*.mp. or exp Egypt/ or egypt*.mp. or egyptian.mp. or exp Egyptian/ or exp Jordan/ or jordan*.mp. or jordanian.mp. or exp Jordanian/ or exp Kuwait/ or kuwait*.mp. or kuwaiti.mp. or exp Kuwaiti/ or exp Lebanon/ or lebanon*.mp. or lebanese.mp. or exp Lebanese/ or exp Libyan Arab Jamahiriya/ or libya*.mp. or exp Libyan/ or libyan.mp. or exp Iran/ or iran*.mp. or exp Iranian people/ or Indo-Iranian people/ or Iranian.mp. or exp Iraq/ or iraq*.mp. or exp iraqi/ or iraqi kurdistan/ or exp Israel/ or israel*.mp. or exp Israeli/ or israeli.mp. or exp Malta/ or malta*.mp. or "Maltese (citizen)"/ or maltese.mp. or exp Morocco/ or morocc*.mp. or exp Moroccan/ or exp Oman/ or oman*.mp. or omani.mp. or exp Omani/ or exp Qatar/ or qatar*.mp. or qatari.mp. or exp Qatari/ or exp Saudi Arabia/ or saudi arabia*.mp. or exp Saudi/ or saudi.mp. or saudi*.mp. or exp Syrian Arab Republic/ or syria*.mp. or syrian.mp. or exp Syrian/ or exp Tunisia/ or tunisia*.mp. or tunisian.mp. or exp Tunisian/ or united arab emirates.mp. or exp United Arab Emirates/ or western asia/ or abu dhabi/ or ajman/ or dubai/ or sharjah/ or exp Emirati/ or emirati*.mp. or UAE.mp. or exp Palestine/ or west bank.mp. or gaza*.mp. or exp Yemen/ or yemen*.mp. or exp Palestinian/ or palestin*.mp. or exp "Gulf of Aqaba"/ or exp Persian Gulf/ or gulf.mp. or hebrew.mp. or "Hebrew (language)"/ or Western Sahara/ or yemeni.mp. or exp Yemeni/ or west asian/ or exp "iranian (citizen)"/

1. **Search strategy in CINAHL (via EBSCOhost Research Databases Interface)**

**Search modes**: Boolean/Phrase

**Expanders:** Apply equivalent subjects

#1**:** physical activit* OR exercise* OR fitness OR walk* OR run* OR sport* OR jog* OR swim* OR sedentar* or resistance train* OR circuit train* OR circuit exercise* OR aerobic train* OR aerobic exercise* OR cardio train* OR HITT OR high intensity interval train* OR step count* OR screen time OR (MH "Screen Time") OR (MH “Television) OR (MH “Sitting”) OR television viewing or screen behavio* or screen watch* or sitting or sit* time or television or TV time.mp. or TV view* or TV watch or watch TV or watch television or view television or prolonged sit* or lying time or physical inactiv* or screen entertainment or screen-based entertainment or video game or video gam* or computer gam* or computer game OR (MH "Exercise+") OR (MH "Physical Activity") OR "exercise" OR (MH "Resistance Training") OR resistance train* OR (MH "Abdominal Exercises") OR (MH "Therapeutic Exercise+") OR (MH "Warm-Up Exercise+") OR (MH "Group Exercise") OR (MH "Sport Specific Training") OR (MH "Open Kinetic Chain Exercises") OR (MH "Aquatic Exercises") OR (MH "Aerobic Exercises+") OR (MH "Upper Extremity Exercises+") OR (MH "Lower Extremity Exercises") OR (MH "Isometric Exercises") OR (MH "Isokinetic Exercises") OR (MH "Back Exercises") OR (MH "Arm Exercises") OR (MH "Anaerobic Exercises") OR (MH "Core Exercises") OR (MH "Isotonic Exercises") OR (MH "Closed Kinetic Chain Exercises") OR (MH "Nordic Walking") OR (MH "High-Intensity Interval Training") OR (MH "Blood Flow Restriction Training") OR (MH "Aerobic Dancing") OR (MH "Life Style, Sedentary+") OR (MH "Video Games") OR (MH "Basketball") OR (MH "Baseball") OR (MH "Walking+") OR weight lift* OR weight train* OR (MH "Weight Lifting") OR (MH "Sports+") OR (MH "Aeronautical Sports") OR (MH "Amateur Sports") OR (MH "Animal Sports+") OR (MH "Aquatic Sports+") OR (MH "Body Building") OR (MH "Bowling") OR (MH "Caving") OR (MH "College Sports") OR (MH "Contact Sports+") OR (MH "Cycling") OR (MH "Endurance Sports") OR (MH "Extreme Sports") OR (MH "Fencing") OR (MH "Golf") OR (MH "Gymnastics") OR (MH "Handball") OR (MH "Martial Arts") OR (MH "Mountaineering") OR (MH "Race Walking") OR (MH "Racquet Sports+") OR (MH "Rock Climbing") OR (MH "Running+") OR (MH "Skating+") OR (MH "Team Sports+") OR (MH "Weight Lifting") OR (MH "Triathlon") OR (MH "Track and Field") OR (MH "Muscle Strengthening+") OR strength train* OR (MH "Endurance Training") OR (MH "High-Intensity Interval Training") OR (MH "Pilates") OR (MH "Plyometrics") OR (MH "Stretching") OR (MH "Callisthenics") OR (MH "Swimming") OR (MH "Triathlon") OR (MH "Jogging")

AND

#2: mobile app* OR mobile phone* OR telemedicine OR telehealth OR mobile health OR mhealth OR m-health OR smartphone* OR mobile technolog* OR text messag* OR short messag* service* OR texting OR mulmedia messag* service* OR activity tracker* OR fitness tracker* OR wearable device* OR wearable technolog* OR smart watch OR smartwatch* OR social media OR cellphone* OR cell phone* OR acceleromet* OR ehealth OR e-health OR whatsapp OR internet (MH "Mobile Applications") OR (MH "Cellular Phone+") OR (MH "Telehealth+") OR (MH "Smartphone") OR (MH “Telemedicine”) OR (MH "Text Messaging+") OR (MH “Fitness Trackers”) OR (MH "Social Media+") OR (MH "Accelerometers") OR (MH "Internet+") OR (MH "Internet-Based Intervention")

AND

#3: middle east* OR islam* OR arab* or north Africa* or north-africa OR africa OR algeria* or Bahrain* or djibouti* or egypt* or jordan* or kuwait* or lebanon* or libya* or iran* or iraq* or israel* or malta* or maltese or morocc* or oman*.mp. or qatar* or saudi arabia* or saudi* or syria* or tunisia* or united arab emirates or emirati* or UAE or Palestin* or west bank or gaza* or yemen* or gulf OR (MH “Middle East+”) OR (MH “Islam”) OR (MH "Arabs") OR (MH "United Arab Emirates") OR (MH "Egypt") OR (MH "Africa, Northern+") OR (MH "Africa+") OR (MH “Algeria”) OR (MH “Bahrain”) OR (MH "Iran") OR (MH "Iraq") OR (MH "Israel") OR (MH "Jordan") OR (MH "Kuwait") OR (MH "Lebanon") OR (MH "Oman") OR (MH "Qatar") OR (MH "Saudi Arabia") OR (MH "Syria") OR (MH "United Arab Emirates") OR (MH "Yemen") OR (MH "Libya") OR (MH "Morocco") OR (MH "Djibouti") OR (MH “Tunisia”)

1. **Search strategy in Scopus**

**Search within:** Article title, Abstract, Keywords

#1:  TITLE-ABS-KEY (“physical activit*” or exercise* or sedentary* or walk* or run* or sport* or “screen behavio*” or “screen time” or “screen watching” or “sitting time” or television or “TV time” or “TV viewing” or “TV watching” or “watch TV” or “watch television” or “view television” or “prolonged sitting” or “lying time” or “physical inactiv*” or “screen entertainment” or “screen-based entertainment” or “video gam*” or “computer gam*”)

AND

#2: TITLE-ABS-KEY (“mobile app*” or “mobile phone*” or “mobile health” or mhealth or m-health or smartphone* or “mobile technolog*” or “text messag*” or “short messag* service*” or texting or “activity tracker*” or “fitness tracker*” or “wearable device*” or “wearable technolog*” or “smart watch*” or smartwatch* or “social media” or cellphone* or “cell phone*” or acceleromet* or ehealth or e-health or internet)

AND

#3: TITLE-ABS-KEY (“middle east*” or islam* or arab* or “north africa*”or “north-africa*” or algeria* or bahrain* or djibouti* or egypt* or jordan* or kuwait* or lebanon* or libya* or iran*or iraq* or israel* or malta* or maltese or morocc* or oman* or qatar* or “saudi arabia*” or Saudi or syria* or tunisia* or “united arab emirates” or emirati* or UAE or “west bank” or gaza or yemen* or Africa* or gulf)

1. **Search strategy in Global Index Medicus**

**Search within:** Title, abstract, subject

**Filters:** Index Medicus for the Eastern Mediterranean region (IMEMR), African Index Medicus (AIM)

#1: (tw:(physical activit* )) or (tw:(exercise*)) or (tw:(sedentar*)) or (tw:(walk*)) or (tw:(run*)) or (tw:(sport*)) or (tw:(screen)) or (tw:(sitting time)) or (tw:(television)) or (tw:(prolonged sitting)) or (tw:(lying time)) or (tw:(physical inactiv*))

AND

#2: (tw:(mobile app*)) OR (tw:(fitness tracker*)) OR (tw:(mobile phone*)) OR (tw:(mobile health*)) OR (tw:(mhealth)) OR (tw:(m-health)) OR (tw:(smartphone*)) OR (tw:(mobile technolog*)) OR (tw:(text messag*)) OR (tw: (short messag* service*)) OR (tw:(texting)) OR (tw:(activity tracker*)) OR (tw:(smart watch*)) OR (tw:(smartwatch*)) OR (tw:(social media)) OR (tw:(cellphone*)) OR (tw:(cell phone*)) OR (tw:(acceleromet*))
