# Supplementary material for: The Use of Mobile Technologies to Promote Physical Activity and Reduce Sedentary Behaviors in the Middle East and North Africa Region: Systematic Review and Meta-Analysis
Source: J Med Internet Res. 2024 Mar 19;26:e53651. doi: 10.2196/53651 (PMC10988381; doi:10.2196/53651)
Supplement: Multimedia Appendix 3 [file jmir_v26i1e53651_app3.docx]

# Appendix 3: Eligibility criteria

| **Domains** | **Inclusion criteria** | **Exclusion criteria** |
| --- | --- | --- |
| Health domain | Physical activity, sedentary behavior | Studies that did not investigate either physical activity or sedentary behavior, including and not limited to: physical function (e.g., rehab after surgery or injury), diet, smoking etc |
| Population | Humans of any age (i.e., including adults, children and adolescents). Both healthy and chronically ill individuals will be included. | Non-human |
| Intervention | The intervention must include any mobile technologies (i.e., mobile apps [37], short message service [SMS], or fitness trackers [20]) as a component to target physical activity and/or sedentary behavior. | Intervention without a mobile technology component; interventions that include only web-based applications or tablet-only applications; pedometers or accelerometers that do not offer continuous access to activity measures; wearable trackers that do not measure physical activity; trackers/apps that are used to measure physical activity only and do not offer any behavioral support. |
| Comparison | - Studies with any control group (e.g., active control, usual care) - Studies without any control group (e.g., qualitative studies) will also be accepted, as we are examining users’ perspectives. | None |
| Outcome | Any measures related to physical activity and/or sedentary behavior. Examples include and are not limited to: daily step number, sitting time.  Since we also examine users’ perspectives, user engagement and acceptability of the interventions will also be examined. Additionally, cognitive or psychological outcomes related to the interventions are also considered (e.g., motivation to change behavior). | Measures related to physical function (e.g., sit-to-stand test). Studies that only reported usage rate of mobile technologies without reporting physical activity outcomes or users’ perspectives will not be considered. |
| Study type | Any primary research study | - Protocols - Opinion pieces/editorials/letters - Review - Conceptual/design/development papers |
| Setting | Studies must have been conducted in the Middle East and North Africa region, which is listed by the World Bank to include the following countries: Algeria; Bahrain; Djibouti; Egypt; Iran; Iraq; Jordan; Kuwait; Lebanon; Libya; Morocco; Oman; Qatar; Saudi Arabia; Syria; Tunisia; United Arab Emirates; West Bank and Gaza; Yemen [2]. | Studies that were conducted outside of the MENA region, as defined previously. |
