# Supplementary material for: The Use of Mobile Technologies to Promote Physical Activity and Reduce Sedentary Behaviors in the Middle East and North Africa Region: Systematic Review and Meta-Analysis
Source: J Med Internet Res. 2024 Mar 19;26:e53651. doi: 10.2196/53651 (PMC10988381; doi:10.2196/53651)
Supplement: Multimedia Appendix 4 [file jmir_v26i1e53651_app4.docx]

# Appendix 4: Calculating the effective sample size of cluster randomized controlled trials for meta-analysis using Cochrane guidelines

The effective sample size of a single intervention group in a cluster randomized trial is its original sample size divided by a quantity called the ʻdesign effectʼ. The design effect is approximately

1 + (M – 1) x ICC

where M is the average cluster size and ICC is the intracluster correlation coefficient.

ICC is assumed to be 0.1, according to Alghafri 2018.

| **Cluster RCTs** | **Average cluster size (M)** | **Design effect** | **Effective sample size (intervention; control)** |
| --- | --- | --- | --- |
| Alghafri 2018 | (82+92)/ (4+4) = 21.75 | 1+ (21.75-1) x 0.1 = 3.075 | Intervention = 82/3.075 ≈ 26  Control = 92/3.075 ≈ 29 |
| Quronfulah 2019 | (32+32)/ (1+1) = 32 | 1+ (32-1) x 0.1 = 4.1 | Intervention = 32/4.1 ≈ 7  Control = 32/4.1 ≈ 7 |
