# Supplementary material for: The Use of Mobile Technologies to Promote Physical Activity and Reduce Sedentary Behaviors in the Middle East and North Africa Region: Systematic Review and Meta-Analysis
Source: J Med Internet Res. 2024 Mar 19;26:e53651. doi: 10.2196/53651 (PMC10988381; doi:10.2196/53651)
Supplement: Multimedia Appendix 5 [file jmir_v26i1e53651_app5.docx]

# Appendix 5: List of excluded studies after full-text review, for not meeting inclusion criteria regarding intervention or outcome

**Wrong intervention**

1. Alanazi, F., et al. (2022). "Poor Compliance of Diabetic Patients with AI-Enabled E-Health Self-Care Management in Saudi Arabia." Information 13(11): 509.
2. Alshehri, F. and F. Alshaikh (2021). "Exploring the constituent elements of a successful mobile health intervention for prediabetic patients in King Saud University Medical City Hospitals in Saudi Arabia: Cross-sectional study." JMIR Formative Research 5(7): e22968.
3. Amer, S. A., et al. (2022). "Prevalence and Determinants of Mobile Health Applications Usage: A National Descriptive Study." Frontiers in Public Health 10: 838509.
4. Arafa, A., et al. (2022). "mHealth App Barriers, Usability, and Personalization: A Cross-Sectional Study from Egypt and Saudi Arabia." Journal of Personalized Medicine 12(12): 2038.
5. Bozorgi, A., et al. (2021). "The effect of the mobile "blood pressure management application" on hypertension self-management enhancement: a randomized controlled trial." Trials [Electronic Resource] 22(1): 1-10.
6. Ghorbani, B., et al. (2021). "Comparing the Effects of Gamification and Teach-Back Training Methods on Adherence to a Therapeutic Regimen in Patients after Coronary Artery Bypass Graft Surgery: Randomized Clinical Trial." Journal of Medical Internet Research 23(12): e22557.
7. Glaser, M., et al. (2022). "The Effects of a Physical Activity Online Intervention Program on Resilience, Perceived Social Support, Psychological Distress and Concerns among At-Risk Youth during the COVID-19 Pandemic." Children 9(11): 1704.
8. Habib-Mourad, C., et al. (2014). "Health-E-PALS: promoting Healthy Eating and Physical Activity in Lebanese school children - Intervention development." Education & Health 32(1): 3-8.
9. Qan'ir, Y., et al. (2021). "Mobile health apps use among Jordanian outpatients: A descriptive study." Health Informatics Journal 27(2): 14604582211017940.
10. Vahedian Shahroodi, M., et al. (2021). "Effect of a theory-based educational intervention for enhancing nutrition and physical activity among Iranian women: a randomised control trial." Public Health Nutrition 24(18): 6046-6057.

**Wrong outcome**

1. Ahmadi, M., et al. (2022). "Development of a Mobile-Based Self-care Application for Patients with Breast Cancer-Related Lymphedema in Iran." Applied clinical informatics 13(5): 935-948.
2. Al-Hamdan, R., et al. (2021). "Efficacy of lifestyle intervention program for Arab women with prediabetes using social media as an alternative platform of delivery." Journal of Diabetes Investigation 12(10): 1872-1880.
3. Al-Hamdan, R., et al. (2019). "Efficacy of different prediabetes program models in improving clinical outcomes in people with prediabetes." Proceedings of the Nutrition Society, FENS 79(OCE2).
4. Etemadifar, S., et al. (2021). "The effects of implementation cardiac rehabilitation program using a mobile application on activity tolerance, fatigue, and dyspnea in patients with myocardial infraction; a randomized clinical trial study." Journal of Isfahan Medical School 38(600): 862-868.
5. Ghorbani, B., et al. (2021). "Comparing the Effects of Gamification and Teach-Back Training Methods on Adherence to a Therapeutic Regimen in Patients after Coronary Artery Bypass Graft Surgery: Randomized Clinical Trial." Journal of Medical Internet Research 23(12): e22557.
6. Zolfaghari, M., et al. (2012). "The impact of nurse short message services and telephone follow-ups on diabetic adherence: which one is more effective?" Journal of Clinical Nursing 21(13-14): 1922-1931.

**Wrong study type**

1. Maddison, R., et al. (2019). "mHealth Interventions for Exercise and Risk Factor Modification in Cardiovascular Disease." Exercise & Sport Sciences Reviews 47(2): 86-90.

**Wrong location**

1. Esentürk, O. K. and E. Yarımkaya (2021). "WhatsApp-Based Physical Activity Intervention for Children With Autism Spectrum Disorder During the Novel Coronavirus (COVID-19) Pandemic: A Feasibility Trial." Adapted Physical Activity Quarterly 38(4): 569-584.
2. O'Brien, T., et al. (2015). "Acceptability of wristband activity trackers among community dwelling older adults." Geriatric Nursing: S21-25.
3. Schwartz, H., et al. (2021). "Staying physically active during the COVID-19 quarantine: exploring the feasibility of live, online, group training sessions among older adults." Translational Behavioral Medicine 11(2): 314-322.
4. Yarimkaya, E., et al. (2022). "A WhatsApp-delivered intervention to promote physical activity in young children with autism spectrum disorder." International Journal of Developmental Disabilities 68(5): 732-743.
