# Supplementary material for: The Use of Mobile Technologies to Promote Physical Activity and Reduce Sedentary Behaviors in the Middle East and North Africa Region: Systematic Review and Meta-Analysis
Source: J Med Internet Res. 2024 Mar 19;26:e53651. doi: 10.2196/53651 (PMC10988381; doi:10.2196/53651)
Supplement: Multimedia Appendix 6 [file jmir_v26i1e53651_app6.docx]

# Appendix 6: Information about funding source and conflict of interest of included studies

| **Author, year** | **Source of funding** | **Conflict of interest** |
| --- | --- | --- |
| Abbaspoor, 2020 | No financial support | None declared |
| Al Ansari, 2023 | None | None declared |
| Al-Daghri, 2022 | The Deanship of Scientific Research, King Saud University | None declared |
| Alghafri, 2018 | Oman Ministry of Health and research council has funded The “MOVEdiabetes” project as part of PhD grants offered to the corresponding author (TSA). Additional funds were provided by The Research Council (TRC) in Oman. | None declared |
| Alghafri, 2020 | Oman Ministry of Health and research council has funded The “MOVEdiabetes” project as part of PhD grants offered to the corresponding author (TSA). | None declared |
| Ali, 2021 | This research was funded by United Arab Emirates University Zayed Center for Health Sciences, 31R059-Research Center-ZCHS-8-2014 and The APC was funded by United Arab Emirates University. | None declared |
| Alnasser, 2019 | The Research and Development Office-Ministry of Education project number (HQI-001) | None declared |
| Alsaleh, 2016 | This research was supported by a doctoral scholarship from Philadelphia University, Jordan (EA). | None declared |
| Alshahrani, 2021 | None | None declared |
| Altabtabaeia, 2021 | Not reported | Not reported |
| Alyousef, 2021 | Not reported | Not reported |
| Ansari, 2022 | All expenses of this study were provided by Ahvaz Jundishapur University of Medical Sciences. | None declared |
| Bardus, 2021 | This research received no external funding. | None declared |
| Chopoghlo, 2021 | This study was a part of a master’s thesis with financial support of IUMS. | None declared |
| Eslami, 2021 | Tabriz University of Medical Sciences | None declared |
| Ghofranipour, 2022 | No funding was obtained for this study. | None declared |
| Goodarzi, 2012 | Not reported | None declared |
| Jorvand, 2020 | None | None declared |
| Khidir, 2021 | Not reported | Not reported |
| Lari, 2018 | Not reported | None declared |
| Parandeh, 2019 | This study was funded by the Research Deputy of Tabriz University of Medical Sciences. | None declared |
| Peyman, 2018 | None | None declared |
| Quronfulah, 2019 | Not reported | Not reported |
| Saleh, 2022 | None | None declared |
| Sani, 2018 | This study was funded by the Medical Research Center, Jizan University, Kingdom of Saudi Arabia. Funding—Project No. JAHIS-1–4. | None declared |
| Yahia, 2021 | No funding sources | None declared |
| Zaman, 2021 | Not reported | None declared |
