# Supplementary material for: The Use of Mobile Technologies to Promote Physical Activity and Reduce Sedentary Behaviors in the Middle East and North Africa Region: Systematic Review and Meta-Analysis
Source: J Med Internet Res. 2024 Mar 19;26:e53651. doi: 10.2196/53651 (PMC10988381; doi:10.2196/53651)
Supplement: Multimedia Appendix 7 [file jmir_v26i1e53651_app7.docx]

# Appendix 7: Retention rates, engagement metrics and other outcomes from experimental studies

**Table S1: Retention rates and other outcomes from included randomized controlled trials**

| **Author, year, country** | **Retention rates (Intervention)** | **Retention rates (Control)** | **Other outcomes or users’ perspectives** |
| --- | --- | --- | --- |
| Ansari, 2022, Iran | 55/55 (100%) in both SMS and app groups | No true control | - Between-group differences in weight, BMI, and waist circumference (p=.001) , favoring the app group over the SMS group (p=.03) - NS: body fat percentage |
| Saleh, 2022, Jordan | 67/76 (88%) | 65/76 (86%) | - Between-group differences in fatigue, shortness of breath, heart failure symptom burden score and quality of life, favoring the intervention (p<.01) - NS: leg or ankle swelling burden scores |
| Alshahrani, 2021, Saudi Arabia | 53/55 (96%) | 50/55 (91%) | - NR other than physical activity outcomes |
| Eslami, 2021, Iran | 70/70 (100%) | 70/70 (100%) | - Between-group differences in the amount of fruits consumed (p=.006), and frequency of consuming fats and oils (p=.002), favoring the intervention - NS: frequency of consuming and amount consumed of bread and cereals, meat, dairy, vegetables, nuts, confections and salt. |
| Abbaspoor, 2020, Iran | 44/50 (88%) | 45/50 (90%) | - Between-group differences in the frequency of consuming of oils and sweets, fruits, vegetables, meat and dairy, favoring the intervention - NS: the frequency of consuming bread and cereals |
| Alghafri, 2020, 2018, Oman | 82/122 (67%) | 92/110 (84%) | - Between-group differences in blood pressure and triglycerides, favoring the intervention - NS: weight, BMI, HbA1c - Almost 90% project officers and 60% participants perceived the intervention as very appropriate and >85% reported that they were satisfied with the program. - Two thirds of participants were likely to recommend the intervention to others. - The consultations, use of pedometers and WhatsApp messages were well perceived by all. - 2/3 of the participants perceived their physical activity behavior to have changed. However, a third (30.4%) were ‘not sure’ or experienced ‘very little’ change or ‘no change’. |
| Parandeh, 2019, Iran | 60/63 (95%) | 61/63 (97%) | - Between-group differences in knowledge (p = .001), perceived susceptibility (p = .001), perceived severity (p = .001), perceived benefits (p = .002), perceived barriers (p = .015), self-efficacy (p = .002), internal cues to action (p = .033) and nutritional performance (p = .029). |
| Quronfulah, 2019, Saudi Arabia | 32/33 (97%) | 32/33 (97%) | - 53.1% of the staff felt that they had managed to reduce sitting at work, while 46.9% felt that they had managed to reduce sitting outside work (at home or with friends). - Most staff reported that they found the text messages and video illustrations of suggested activities during breaks were useful at work (both: 81.3%) and less so outside work (78.1%, 71.9%, respectively). - More than half of the staff found the prompting software useful at work (62.5%). However, the majority of staff (90.6%) were neutral or disagreed that the prompting software was useful for changing their behavior outside work. |
| Alsaleh, 2016, Jordan | 66/71 (93%) | 79/85 (93%) | - Between-group differences in blood pressure, weight, BMI, quality of life and self-efficacy for exercise (p<.05), favoring the intervention - 100 % reported that they valued all elements of the intervention, including goal-setting, self-monitoring and feedback and delivery methods including face-to-face consultation, telephone call consultations and text messages. - Participants reported several benefits of the intervention including: gaining knowledge about how to make behavioral changes, receiving regular reminders and encouragement which motivated them to be more active, and feeling supported by and building a trusting relationship with the cardiac nurse. - 20% (13/66) mentioned barriers to being active after the intervention, including poor health, not having enough time and not enjoying physical activity. |
| Goodarzi, 2012, Iran | 43/50 (86%) | 38/50 (76%) | - Between-group differences in HbA1C (p=.024), LDL-C (p=.019), total cholesterol (p=.002), BUN, micro albumin, knowledge, practice and self-efficacy (p≤.001) |

**Abbreviations:** BMI: Body Mass Index; BUN: blood urea nitrogen; HbA1c: Hemoglobin A1C; LDL: low-density lipoprotein cholesterol; NS: not significant; SMS: Short Message Service

**Table S2: Retention rates and engagement metrics from included quasi-experimental studies**

| **Author, year, country** | **Intervention engagement** | **Retention rates (Intervention; control *if applicable)** |
| --- | --- | --- |
| Al-Daghri, 2022, Saudi Arabia | NR | 643/2600 (25%) |
| Ghofranipour, 2022, Iran | 3/13 mothers reported watching all videos; 2 did not watch the videos. | 13/13 (100%) |
| Ali, 2021, UAE | NR | 54/61 (89%)  Control: 111/185 (60%) |
| Alyousef, 2021, Saudi Arabia | NR | NR |
| Chopoghlo, 2021, Iran | NR | NR |
| Khidir, 2021, Qatar | In the 1^st^ phase, 155 participants used mobile apps; 133 used pedometers. In the 2^nd^ phase, 55 used mobile apps and 54 used pedometers. This perhaps suggests that participants preferred to use mobile apps to measure steps than pedometers.  The online system monitored the steps uploading actions performed by the users throughout  both phases; a number of 23.378 uploads were observed in 2018, while in 2019 the subjects uploaded the data 9417 times. The difference between the number of uploads  in phase 1 and phase 2 perhaps indicated that the first group, the one that received  uploads reminders, educational information about healthy lifestyle and benefits of walking during the  walking intervention in 2018 were more likely to upload their step count. | NR |
| Yahia, 2021, Egypt | NR | 100% |
| Jorvand, 2020, Iran | NR | 100%  Control: 100% |
| Alnasser, 2019, Saudi Arabia | Users had to use the app at least once every 2 weeks to be considered ‘engaged’. At 2 months, 21/66 (31.8%) were considered engaged users. At 6 months, 26/47 (55.3%) were considered engaged users. | 42/240 (17.5%) |
| Lari, 2018, Iran | NR | 37/40 (93%)  Control: 36/40 (90%) |
| Peyman, 2018, Iran | NR | 180/180 (100%)  Control: 180/180 (100%) |
| Sani, 2018, Saudi Arabia | NR | NR |

NR: not reported
