# Supplementary material for: The Use of Mobile Technologies to Promote Physical Activity and Reduce Sedentary Behaviors in the Middle East and North Africa Region: Systematic Review and Meta-Analysis
Source: J Med Internet Res. 2024 Mar 19;26:e53651. doi: 10.2196/53651 (PMC10988381; doi:10.2196/53651)
Supplement: Multimedia Appendix 8 [file jmir_v26i1e53651_app8.docx]

# Appendix 8: Funnel plot of standard error by standardized mean difference


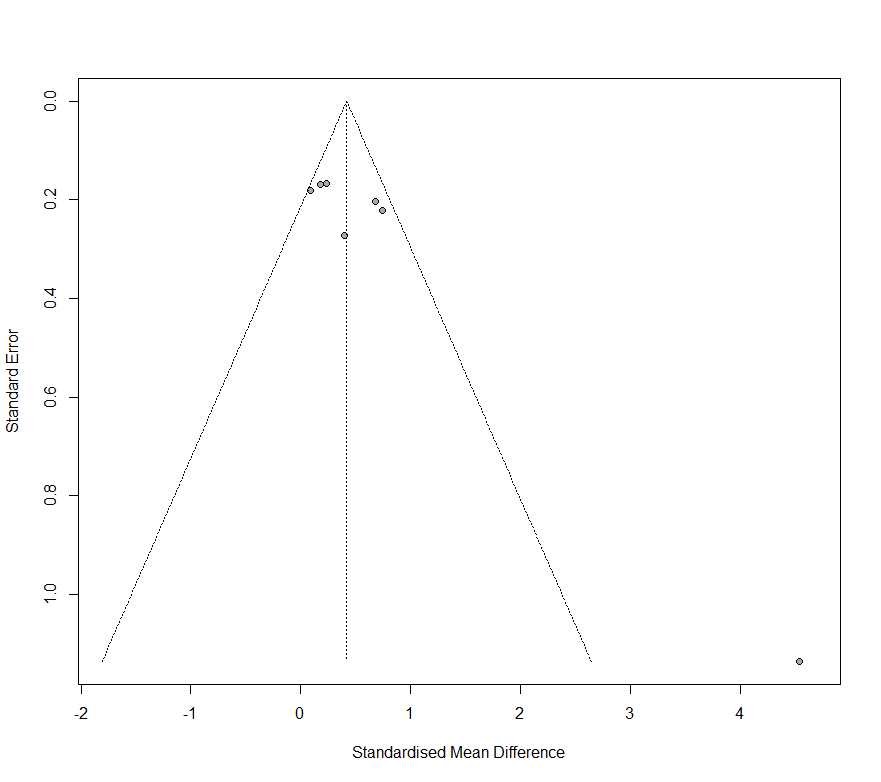


The funnel plot appeared to indicate signs of publication bias. The Egger’s test had an intercept of 3.65 (p=.015), indicating possible publication bias. The Trim and Fill method imputed an effect size of 0.30 (95% CI -0.65 to 1.25).
