# Supplementary material for: The Use of Mobile Technologies to Promote Physical Activity and Reduce Sedentary Behaviors in the Middle East and North Africa Region: Systematic Review and Meta-Analysis
Source: J Med Internet Res. 2024 Mar 19;26:e53651. doi: 10.2196/53651 (PMC10988381; doi:10.2196/53651)
Supplement: Multimedia Appendix 9 [file jmir_v26i1e53651_app9.docx]

# Appendix 9: Sensitivity analyses and subgroup analyses

**Sensitivity analyses**

| **Description** | **Number of studies** | **Standardized difference in means (95% CI)** | **I^2^ (%)** | **P-value** |
| --- | --- | --- | --- | --- |
| including studies that were randomized at an individual level | 5 | 0.36 (0.11, 0.62) | 58.3 | .005 |
| Including studies with 3 or more low risk of bias categories | 3 | 0.24 (0.03, 0.45) | 0 | .03 |
| Excluding one study (i.e., Quronfulah) that had results as an outlier | 6 | 0.36 (0.14, 0.58) | 47.9 | .001 |

CI: confidence interval

**Subgroup analyses**

| **Description** | **Number of studies** | **Standardized difference in means (95% CI)** | **I^2^ (%)** | **P-value** |
| --- | --- | --- | --- | --- |
| including studies that had participants with a chronic condition | 2 | 0.28 (-0.0005, 0.56) | 0 | .05 |
| Including studies that had healthy participants | 5 | 1 (-0.26, 2.28) | 97.53 | .12 |

CI: confidence interval
